# Let Nature Be Thy Medicine: A Socioecological Exploration of Green Prescribing in the UK

**DOI:** 10.3390/ijerph17103460

**Published:** 2020-05-15

**Authors:** Jake M. Robinson, Anna Jorgensen, Ross Cameron, Paul Brindley

**Affiliations:** 1 Department of Landscape, University of Sheffield, Sheffield S10 2TN, UK; a.jorgensen@sheffield.ac.uk (A.J.); R.W.cameron@sheffield.ac.uk (R.C.); p.brindley@sheffield.ac.uk (P.B.); 2 inVIVO Planetary Health, of the Worldwide Universities Network (WUN), West New York, NJ 10704, USA; 3 Healthy Urban Microbiome Initiative (HUMI), Adelaide, SA 5005, Australia

**Keywords:** green prescriptions, planetary health, nature-based interventions, urban nature, biodiversity, mental health, nature connectedness, greenspace, noncommunicable diseases, upstream health interventions

## Abstract

Prescribing nature-based health interventions (green prescribing)—such as therapeutic horticulture or conservation activities—is an emerging transdisciplinary strategy focussed on reducing noncommunicable diseases. However, little is known about the practice of, and socioecological constraints/opportunities associated with, green prescribing in the UK. Furthermore, the distribution of green prescribing has yet to be comprehensively mapped. In this study, we conducted a socioecological exploration of green prescribing. We deployed online questionnaires to collect data from general practitioners (GPs) and nature-based organisations (NBOs) around the UK and conducted spatial analyses. Our results indicate that GPs and NBOs perceive and express some common and distinct constraints to green prescribing. This highlights the need to promote cross-disciplinary communication pathways. Greenspace presence and abundance within close proximity (100 and 250 m) to GP surgeries (but not greenness—as a proxy for vegetation cover) and NBO presence within 5 km were associated with higher levels of green prescribing provision. Lower levels of deprivation were associated with higher frequency of NBOs. This suggests that the availability of greenspaces and NBOs could be important for green prescribing provision, but there could be greater opportunities in less deprived areas. Important foci for future research should be to establish transdisciplinary collaborative pathways, efficient infrastructure management and a common vocabulary in green prescribing—with the overall aim of reducing inequalities and enhancing planetary health.

## 1. Introduction

It is now widely accepted that spending time in natural or semi-natural environments (e.g., forests, grasslands, gardens and parks) can result in significant positive mental and physical health benefits [1,2,3]. For example, the Japanese practice of Shinrin-yoku or ‘forest bathing’ has been shown to enhance innate immunity via lymphocyte cell activity and can reduce diastolic and systolic blood pressure [4,5]; gardening can provide relief from acute stress and improve symptoms of depression [6,7]; and simply spending time in nature can enhance psychological restoration (the ability to recover from stress) and can facilitate healthy child development [8,9,10]. Through the biophilia hypothesis, Wilson (1984) argues that humans hold an innate affinity to seek connections with nature. Furthermore, there is evidence to suggest that the environmental microbiome—the diverse consortium of microorganisms in a given environment—can have positive developmental and regulatory influences on the immune system and potentially anxiolytic effects [11,12,13]. This latter claim is supported by a recent mouse study, in which exposure to trace levels of biodiverse soil dust was significantly associated with reduced anxiety like behaviours [13]. Natural environments can also provide important places for reflection and introspection, for cultivating feelings of awe, inspiration and freedom, and for facilitating group-based convivial activities, which could help to improve social cohesion and enhance mental health [14,15,16,17].

Interacting with nature for salutogenic effects is by no means a novel concept. From a Western societal perspective, the fundamental principles of nature-based therapies can be traced back to the Hippocratic era (460–370 BC), when changing environments and lifestyle practices were advised by the physicians of the time [18]. Furthermore, the Greeks and Romans established thermal spa baths to improve health and well-being [19,20,21]. From a traditional ecological knowledge perspective, indigenous Australians recognised the deep connections between mental and physical health and the “land and river”, and Canadian First Nations’ holistic view of health highlights the interrelatedness of human well-being and the environment [22,23]. It is important to recognise that our complex societies have evolving views, social behaviours and health-related needs, and it is unrealistic to view spending ‘time in nature’ as a panacea—i.e., it is unlikely to be suitable for everyone and for all conditions.

However, there is growing interest in ‘green prescribing’ (GRx) as a contemporary practice of prescribing nature-based health interventions, particularly for noncommunicable diseases [24,25,26]. Green prescribing builds on the earlier concept of prescribing exercise and diet-based interventions [27]—a variant that was pioneered by general practitioners (GPs) in New Zealand in the 1990s [28]. It also builds on the recent social prescribing movement, which can be defined as: “a way of linking patients in primary care with sources of support within the community—usually provided by the voluntary and community sector, offering GPs a non-medical referral option that can operate alongside existing treatments to improve health and well-being”, [29] (p. 7) [30,31].

Green prescriptions are typically administered to patients with a defined need and can be used to complement orthodox medical practices [32,33]. Nature-based intervention activities can include therapeutic horticulture, biodiversity conservation activities, care farming (i.e., farming practices for health, socialising and education), nature walks, and social activities in greenspaces [34,35,36]—and although the social element is often important, it is not a necessity. To establish effective and sustainable green prescribing schemes, cooperative interactions between primary care professionals and nature-based organisations (NBOs) are typically required, and the ability to speak multiple disciplinary ‘languages’ is considered an essential asset [37].

There is potential for green prescribing to contribute to health care (reactive) and sustainable health promotion (proactive), while potentially bringing important co-benefits (e.g., social, environmental, and economic benefits) [38]. However, it is still an emerging and unorthodox strategy. As such, initial adoption may be sporadic and limited. In the UK, little is known about the status of (distribution and practice), and socioecological constraints and opportunities associated with green prescribing. To our knowledge, no one has explicitly mapped nationwide green prescribing services/infrastructure. To this end, mapping could be a useful policy action (e.g., for informing targeted resource allocation). Moreover, gaining insights into the perceived constraints of green prescribing from the view of primary care professionals and NBOs could help to synchronise knowledge and empathy and identify disciplinary barriers to aid in future management and delivery. Furthermore, exploring ecological, spatial and social factors that may affect green prescribing could also provide important insights for policy makers.

In this study, we conducted a socioecological exploration of the green prescribing health intervention model in the UK. Our primary aims for this study were to (a) explore awareness, constraints and opportunities associated with green prescribing, focusing on general practitioners (as potential prescribers) and nature-based organisations (as potential providers) around the UK; (b) collect spatial data to estimate the general distribution of green prescribing; and (c) to explore whether available services, geography, greenspace, and deprivation influenced green prescribing awareness, provision and constraints. 

## 2. Materials and Methods

### 2.1. Online Questionnaire and the Web-Scrape Process

We formulated two online-based questionnaires—one for GPs (as potential service prescribers) and one for nature-based organisations (as potential service providers). The questionnaires included 8–10 structured questions, formulated with the aid of a pilot study and a group of GP volunteers prior to commencing the research. The questionnaires were ethically reviewed by the University of Sheffield’s Department of Landscape internal review committee and by the National Health Service’s (NHS) Health Research Authority (HRA); Integrated-Research Application System (IRAS) reference number: 261514. 

**Figure A1:**
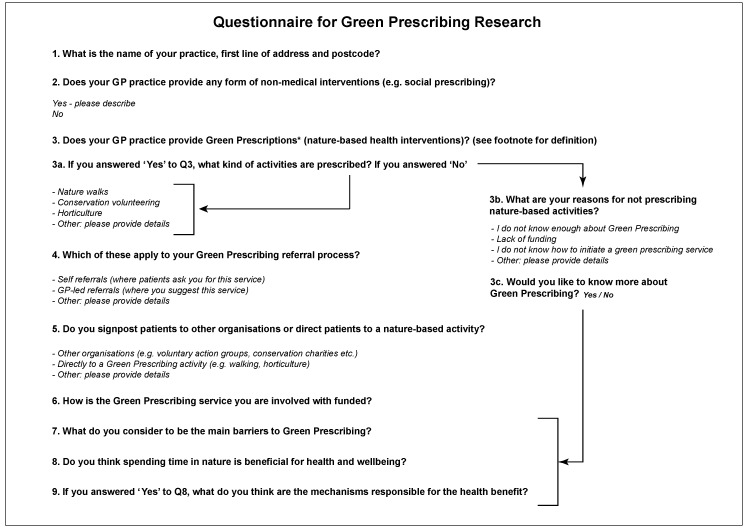
Green prescribing questionnaire for GPs.

**Figure A2:**
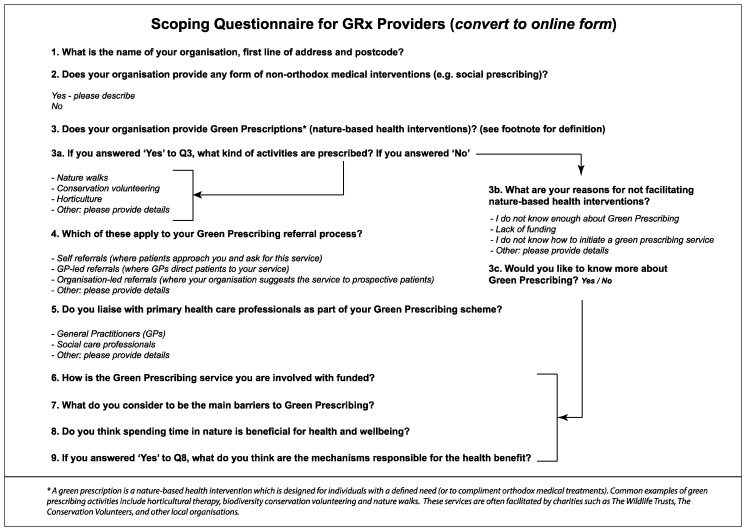
Green prescribing questionnaire for NBOs.

The research questionnaires included key questions regarding geolocation, awareness and status of green prescribing, and a question to ascertain what the respondents considered to be the main constraints to green prescribing. The questions are set out in Figure A1 and Figure A2 in Appendix A.

The online questionnaires were distributed to GPs and NBOs across the UK (between March and September 2019) via an introductory email with a detailed participant information sheet, consent form and a secure link to the questionnaire. The questionnaires were hosted by the University of Sheffield’s Google Forms account. Contact details for the GPs were obtained via the publicly-available NHS online contact directory (www.nhs.uk/service-search/find-a-gp) and by contacting the Clinical Commissioning Groups (CCG) directly. The protocol for approaching GPs was also ethically reviewed by the HRA.

The contact details for the NBOs were obtained via a web-scrape process (web data searched and copied into a central local database) combined with an approach based on the Preferred Reporting Items for Systematic Reviews and Meta-Analyses (PRISMA) workflow [39,40]. 

**Figure 1:**
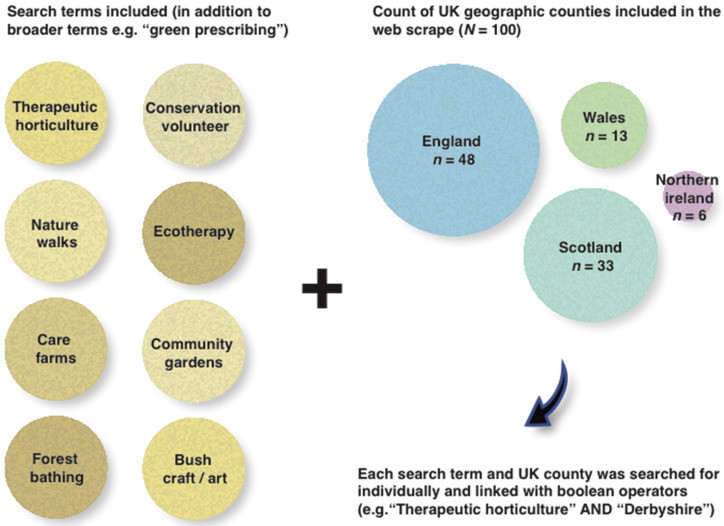
Green prescribing web scrape search method for nature-based organisations. Search terms are shown on the left, and a count breakdown of UK counties per country on the right.

To obtain a list of all the relevant organisations either currently facilitating or having the potential to facilitate green prescribing schemes in the UK, a set of relevant search terms were compiled (e.g., “green prescriptions”; “green care”; “nature-based intervention”). These were then tested and refined in the Google search engine and filters were applied to include only UK results. Additionally, green prescribing activity search terms were used for each of the 100 geographic counties (subnational divisions) in the UK (Figure 1). Where possible, email contact details were obtained and geographic coordinates were acquired for subsequent GIS analysis. 

A detailed participant information sheet and informed consent form was also provided to the nature-based organisations. Once the responses were entered and submitted, they were downloaded by the researchers in a comma separated values (.csv) format for subsequent processing and analysis. The questionnaire structure and plan for maximizing response rate was informed by references [41,42,43]. 

### 2.2. Coding of Open-Ended Responses

For the perceived barriers question (Q.7 Figure A1 in Appendix A), the open-ended response format was chosen to allow respondents to “use their own language and express their own views” [44] (p. 9). To classify and “clean” the data for subsequent analysis, the responses to the questions with the open-ended answer format (descriptive) were coded. 

The approach to interpret these textual responses was to read through each answer several times in a spreadsheet, seeking key recurring themes. These themes specifically related to the focal topics and respondent views. A set of theme codes were generated, providing “the basis for surfacing the frequency of occurrence of themes” in preparation for subsequent quantitative analysis [44] (p. 29). A short and perfunctory response or more in-depth response could be assigned the same code—for example, “lack of funding” and a detailed response with an obvious focus on the lack of funding would be given the code ‘Funding’ (as a key constraint to green prescribing). 

### 2.3. GIS Data

Once the spreadsheets containing the responses and geolocations were cleaned, they were saved as .csv files and imported into QGIS 3.4 as vectors layers. These were then converted to ESRI point shapefiles. 

#### 2.3.1. Buffer Analysis

The point files were separated into four categories, as follows: “Yes” to green prescribing provision (responses from GPs); “No” to green prescribing provision (responses from GPs); “Yes” to green prescription facilitation (responses from NBOs); “No” to green prescription facilitation (responses from nature-based organisations). 

**Figure 2:**
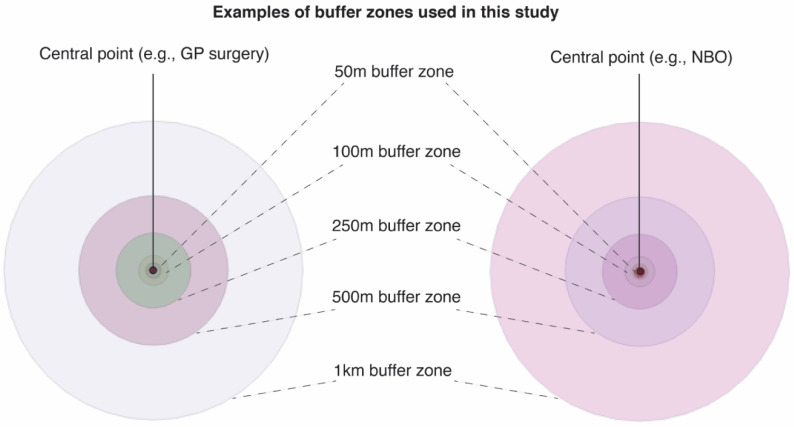
Example of buffer zones created around each point file containing attribute data (spatial information and questionnaire responses) for GPs and nature-based organisations in the UK.

Using vector geoprocessing tools, circular buffer zones (radii from central coordinate of GP surgery or NBO) of 50 m, 100 m 250 m, 500 m, 1 km and 5 km were then created around each point to facilitate spatial analyses (Figure 2). These radii have been used in several spatial studies involving the built environment, urban green spaces and human health [45,46,47].

#### 2.3.2. Graduated Symbology

To provide map outputs and descriptive statistics of the web-scrape results, UK county boundary datasets were obtained from UK government sources (e.g., https://ckan.publishing.service.gov.uk/dataset and https://opendatani.gov.uk/dataset). 

Green prescribing activity attributes were then joined ‘by location’ to the county boundary datasets using vector data management tools. The symbologies were subsequently graduated and classified to provide a visual representation of quantitative differences in values using defined colour ramps. 

#### 2.3.3. Landscape/Environmental Datasets

To analyse aspects of greenspace and infrastructure, the OS Open Greenspace dataset (a comprehensive dataset of publicly accessible urban greenspaces) was imported into QGIS as a polygon vector layer with a point layer for greenspace access locations. These datasets have been used in several urban greenspace studies [48,49].

A measure of greenness (mean greenness for each buffer zone) was also calculated using NASA Landsat 8 Imagery (30 m resolution), isolating spectral bands 4 (Red) and 5 (Near Infrared) and applying the equation for the Normalised Difference Vegetation Index (NDVI). This process provides a score of estimated landcover greenness, where 0 represents no greenness and 1 represents high levels of greenness—used as a proxy for vegetation cover. The equation to obtain this metric is as follows:

$$\frac{Near\;Infrared\;Light-Red\;}{Near\;Infrared\;Light+Red\;}$$

Using the Raster algebraic expression calculator, the above equation was applied to the two spectral band layers, i.e., Red and Near Infrared (NIR). The resulting outputs were subsequently rendered into a single band pseudocolour and represented using a RdYlGn (Red-Yellow-Green) colour ramp.

#### 2.3.4. Deprivation Data

**Figure 3:**
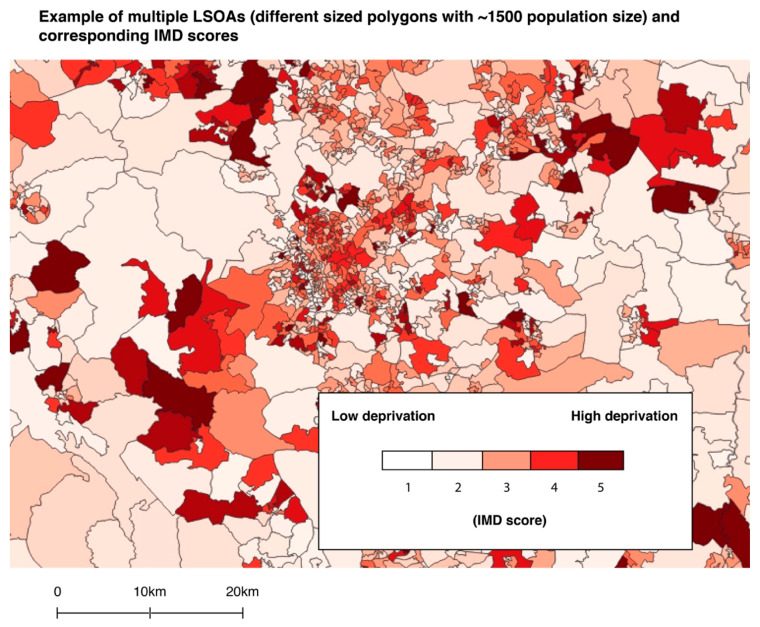
Example of Lower Super Output Areas (LSOAs) (boundaries) with Index of Multiple Deprivation (IMD) scores using ‘categorised’ symbology in QGIS.

To explore relationships between green prescribing and deprivation, quintile scores from an Index of Multiple Deprivation (IMD) dataset previously adjusted for each UK country was used [50]. IMD data have been used in several greenspace epidemiology studies [51,52,53]. The IMD provides multivariate data on relative deprivation in Lower Super Output Areas (LSOAs) for England, Wales and Northern Ireland and data zone layers for Scotland (Figure 3). LSOAs are boundary areas containing an average population of approximately 1500 and up to 1000 in data zones. These geographic boundaries have been used in similar socioecological studies [54,55,56].

### 2.4. Spatial and Statistical Analyses

To facilitate quantitative analysis and maximise UK-wide representation, the aim was to acquire *n* = 367 responses from GPs based on an approximate population size (of UK GP practices) of 8000 [57], with a 95% Confidence Level and a 5% Margin of Error. For NBOs, the aim was to acquire a sample size of *n* = 251. This was based on the *n* = 714 results from the web-scrape, with a 95% Confidence Level and a 5% Margin of Error. 

To facilitate quantitative analysis of potential relationships between the presence or absence of green prescriptions and the independent variables (e.g., greenspaces; deprivation etc.), the ‘Yes’/’No’ questionnaire responses for Question 3 (i.e., “Does your GP practice provide green prescriptions?”) were extracted and recoded to numerical binary variables, where 1 = Yes/Present; and 0 = No/Absent. 

We used a combination of parametric and nonparametric statistical tests and qualitative coding to facilitate the analyses. 

#### 2.4.1. Landscape and Environmental Metrics

##### OS Open Greenspace

To determine whether the presence (and count) of greenspaces within (and touching, i.e., greenspaces partially in the buffer zone were included) a certain radius of GP surgeries was associated with green prescribing provision, the OS Open Greenspace dataset and the georeferenced binary responses for Question 3 were imported into QGIS. The greenspace polygons within each buffer zone (50 m, 100 m, 250 m, 500 m, 1 km and 5 km) were extracted and counted using vector data management tools. The joined data were then exported to a .csv file for subsequent statistical analysis in the R statistical computing environment via the R Studio interface version 1.2.1335.

Due to the nonnormal (right skew) distribution of the samples, nonparametric statistical tests were selected. A Mann–Whitney U test was conducted to explore differences between the number of greenspaces within 100 and 250 m of the GP surgeries that provided green prescribing vs. GP surgeries that did not provide them (500 m and 1 km radii were excluded due to no relationships for these ranges, and the 50 m buffer was excluded due to an absence of greenspaces within this radius). 

##### NDVI

**Figure 4:**
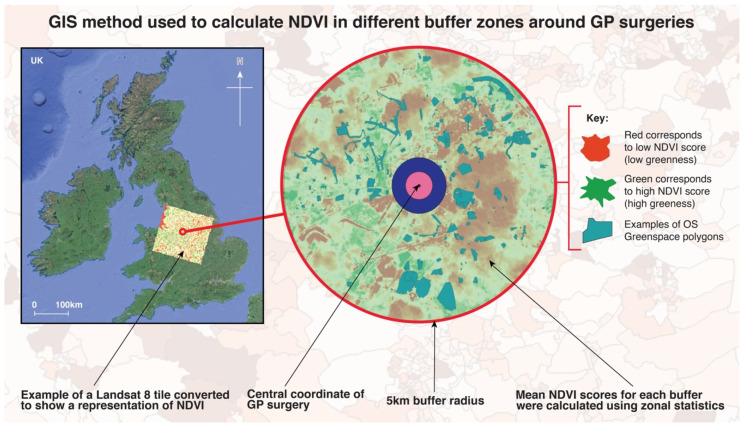
Example of buffer zones around GP surgeries with a visual representation of the Normalised Difference Vegetation Index (NDVI). The mean values within these buffers was calculated and exported for further analysis. The whole of the UK was overlaid with the NASA Landsat 8 tiles to facilitate NDVI calculations.

For the NDVI analysis, firstly we reprojected the vector (buffer) layers to match the coordinate reference system (CRS) of the Landsat 8 raster files and then calculated the mean NDVI values for all buffer zones using the zonal statistics raster analysis tool (Figure 4). The updated attribute table was exported as a .csv file for subsequent statistical analysis. 

Once the mean NDVI scores were calculated, a binomial logistic regression model was used to predict whether mean NDVI (a representation of greenness) in each buffer zone had a significant influence over the binary dependent variable (where 1 = “Yes” to represent the GPs that do provide nature-based interventions; and 0 = “No” to represent the GPs that do not provide nature-based interventions). 

#### 2.4.2. Deprivation

For the analysis of deprivation, UK quintile scores from 1 (lowest deprivation) to 5 (highest deprivation) were extracted from the adjusted IMD dataset. These scores were joined to each LSOA and data zone and used for subsequent analysis. To explore whether deprivation influenced the provision of nature-based interventions, Mann–Whitney U tests were conducted. This approach was suitable for comparing IMD scores between the four variables (GPs that did and did not prescribe GRx; and NBOs who did and did not provide GRx). 

To test whether a relationship existed between levels of deprivation and NBO presence, we joined the web-scrape results for NBOs with the UK IMD and boundary datasets. We subsequently conducted Chi Sq (X^2^) tests to compare expected vs. actual observations. This test provided what the probability was that differences in values (frequency of observations) are by chance under the assumption of independence.

#### 2.4.3. Nature-Based Organisation Presence and GRx Provision

We also tested whether presence of NBOs was associated with provision of GRx by GP surgeries. For this element we explored the potential incidence of the NBOs confirming GRx facilitation (from the questionnaire responses) and also data from the web-scrape of NBOs (*n* = 714). We used a Mann–Whitney U test and a 2-sample test for equality of proportions.

## 3. Results

### 3.1. Descriptive Statistics

A total of *n* = 284 respondents completed the research questionnaire. The number of GPs participating in the study was *n* = 114 (from *n* = 211 CCGs and *n* = 625 individual practices). The Confidence Level and Margin of Error for this sample size are 95% and 9%, respectively. For NBOs (from *n* = 714 identified by the manual web-scrape), a total of *n* = 170 responded. The Confidence Level and Margin of Error for this sample size are 95% and 6.6%, respectively. The majority of responses came from England-based practices and organisations.

#### 3.1.1. Results from the Questionnaire (Presence/Absence of Green Prescription Provision)

**Figure 5:**
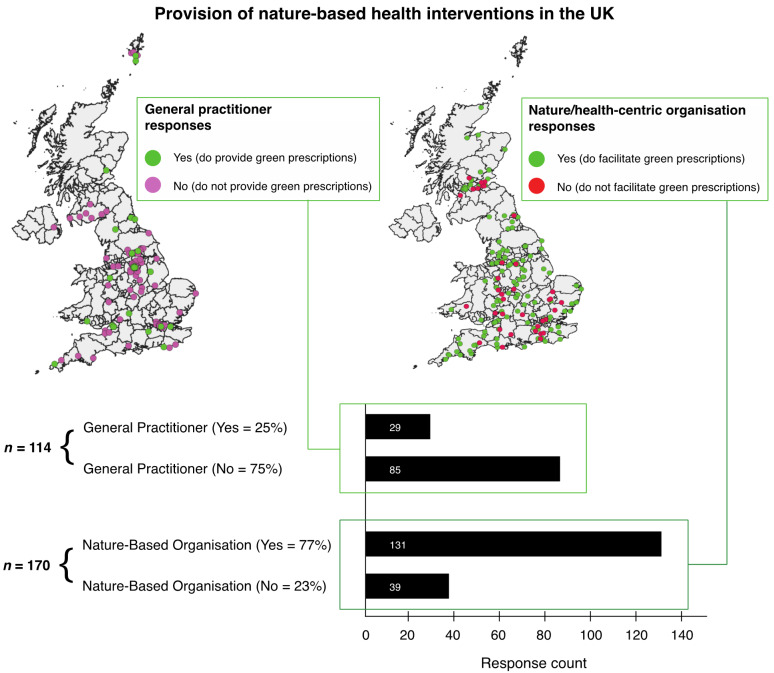
Provision of nature-based health interventions (green prescriptions) in the UK based on the questionnaire responses. This figure shows the location and distribution of responses to the question “*Does your GP surgery provide green prescriptions?*” (or a similar question for nature-based organisations).

Based on the count of questionnaire responses by GPs, *n* = 29 GPs did prescribe nature-based interventions and *n* = 85 GPs did not. In terms of NBO responses, *n* = 131 did provide (i.e., facilitate activities) nature-based interventions and *n* = 39 did not (Figure 5). 

#### 3.1.2. Results from the Coding of the Perceived Constraints Question (for GPs)

The results of the analysis of what GPs perceive as key constraints to green prescribing showed that ‘available services’ (organisations and processes that facilitate nature-based interventions) was mentioned the most frequently by GPs (*n* = 33). Funding for the service and awareness of the green prescribing concept were also frequently mentioned (*n* = 31 and *n* = 29, respectively). However, we are unable to confirm whether ‘awareness’ refers to GPs, patients or both.

**Figure 6:**
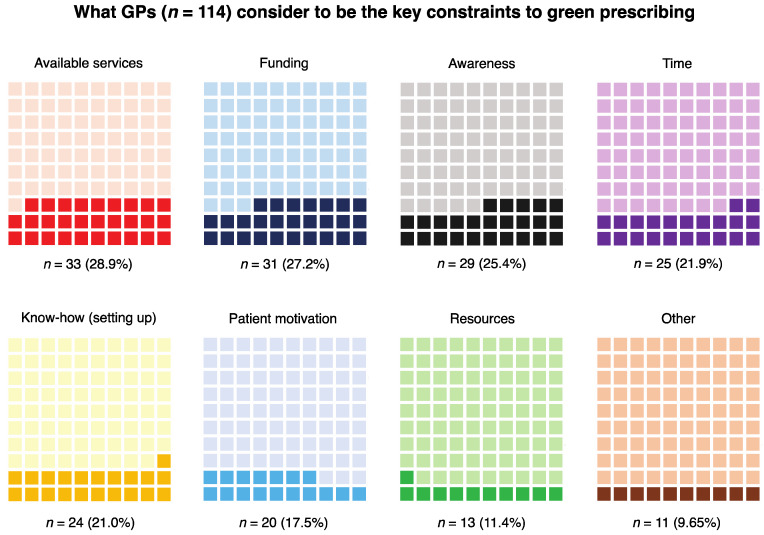
Waffle charts showing what GPs consider to be the key constraints to green prescribing. These charts show proportions with actual response counts and corresponding percentages below. The proportions are presented in descending order (i.e., of response frequency) from top left to bottom right.

Time constraints (*n* = 25) (note: there is an assumption here that this refers to GP time), ‘know-how’ (i.e., knowledge of how to set up a green prescribing service) (*n* = 24), patient motivation (and confidence to attend the interventions) (*n* = 20), and having the appropriate resources to provide a green prescribing service (this could overlap somewhat with time and funding) (*n* = 13) were also mentioned by several GPs (Figure 6). 

#### 3.1.3. Results from the Coding of the Perceived Constraints Question (for Nature-Based Organisations)

The results of the analysis of what NBOs perceive as key constraints to green prescribing showed that funding (i.e., the organisations typically have small financial budgets) was the most frequently mentioned constraint (*n* = 86). Awareness and understanding of the benefits of spending time in nature were also conveyed as important constraints several times by NBOs (*n* = 30 and *n* = 38, respectively). It is likely that these constraints are aimed at GPs and potentially also patients as the responses suggest that, in general, NBOs are aware of the potential benefits.

**Figure 7:**
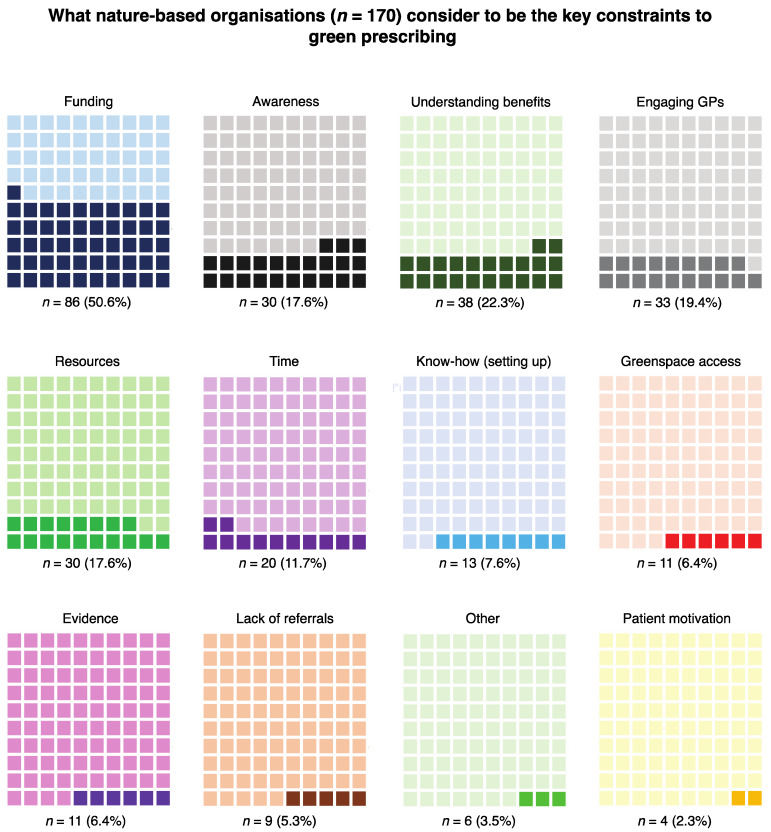
Waffle charts showing what nature-based organisations consider to be the key constraints to green prescribing. These charts show proportions with actual response counts and corresponding percentages below. The proportions are presented in descending order (i.e., of response frequency) from top left to bottom right.

Distinctively NBO-based themes included engaging GPs (*n* = 33) (some respondents suggest it is difficult to “engage the NHS at all levels, and disseminating information through the NHS can [also] be difficult”, and GPs are “not able or willing to green prescribe”), greenspace access (*n* = 11) (e.g., landowner permission, transport costs, but also some people are “house bound”), green prescribing ‘referrals’ which could be synonymous with engaging GPs (*n* = 9), and ‘evidence’ to support benefits of green prescribing (*n* = 11) (some respondents feel there is still not a strong enough evidence base to persuade health professionals to engage in the interventions) (Figure 7). 

#### 3.1.4. Results from the Web-Scrape Process (for Nature-Based Organisations)

**Figure 8:**
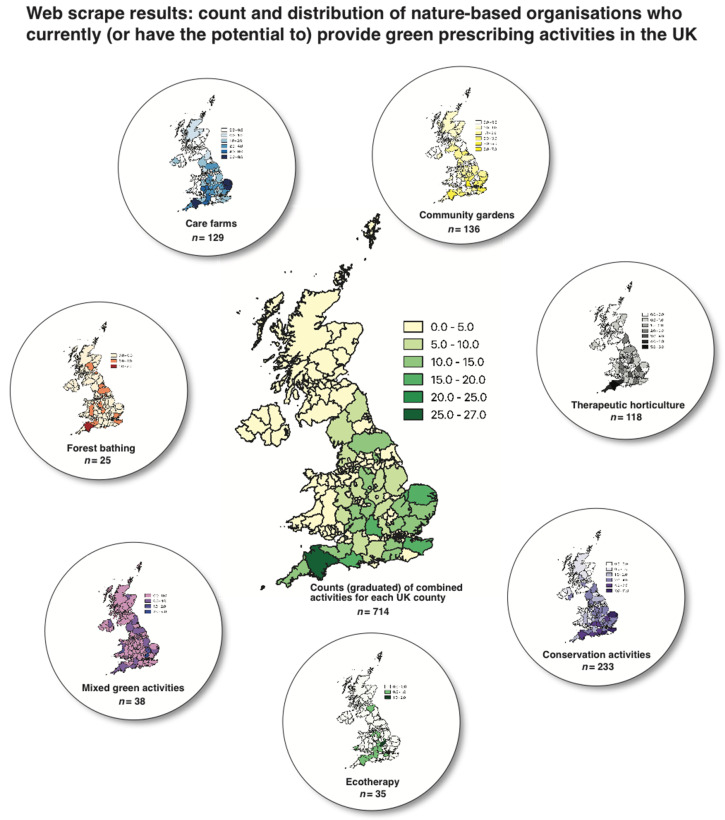
UK map of counties showing count (*n* = 714) and distribution of nature-based organisations which currently (or have the potential to) provide green prescribing activities (inlets show counts/distribution for individual activities). The quantitative differences in values are presented using graduated symbology and an appropriate colour ramp. This was processed in QGIS.

The web-scrape resulted in the acquisition of *n =* 714 NBOs who either provided green prescribing activities or had the potential to do so based primarily on organisation/service type. These fall into seven themes including care farms (*n =* 129), community gardens (*n* = 136), therapeutic horticulture (*n* = 118), conservation activities (*n* = 233), ecotherapy (*n =* 35), mixed green activities (such as bush crafts and walks; *n =* 38), and forest bathing (*n =* 25) (Figure 8). 

Conservation activities/organisations returned the highest number of records (*n =* 233) and forest bathing the lowest (*n =* 25). There are clear differences between the number of advertised NBOs in England (i.e., more abundant) compared to Northern Ireland, Scotland and Wales. Zero records were returned for several UK counties (e.g., Kincardineshire in Scotland), whereas *n =* 27 records (the highest number) were returned for Devon in the southwest of England. 

### 3.2. Results from Spatial and Inferential Statistical Analyses

The following section presents the results from both the spatial analyses conducted in QGIS using landscape/environmental and sociological (deprivation) datasets and the statistical analyses carried out primarily in the R statistical computing environment. 

#### 3.2.1. Landscape and Environmental Metrics

**Figure 9:**
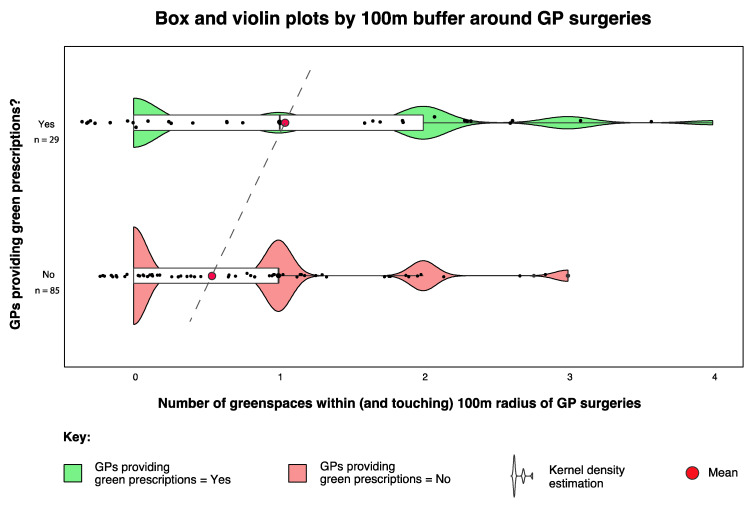
Boxplot showing differences in greenspace abundance within the 100 m buffer zone around GP surgeries that did (green) and did not (red) prescribe nature-based interventions. The maximum number within 100 m of any practice was *n =* 4. The violin plots show kernel density estimation, i.e., representing the distribution shape of the data and the points have a small amount of random variation (jitter) to reduce over-plotting.

The data for greenspace presence within different buffer zones around GP surgeries were found to have nonnormal (right skew) distributions. Therefore, nonparametric tests were used for statistical analysis. We conducted a Wilcoxon rank sum test with continuity correction and found that mean greenspace abundance within 100 m of group 1 (GPs prescribing nature-based interventions; $\bar{x}$ = 1.17) was significantly different (greater) to the same radius for group 2 (GPs not prescribing nature-based interventions; $\bar{x}$ = 0.51) (W = 853, *p =* 0.005) (Figure 9).

A 2-sample test for equality of proportions also confirmed that a greater proportion of GPs who prescribed nature-based interventions had a greenspace present within (including partial intersect) 100 m radius (17 out of 29 or 58.68%) compared to those who did not (31 out of 85 or 36.4%) (X-squared = 5.05, df = 1, *p =* 0.047). The same analysis but for greenspaces fully within the 100 m radius buffer (6 out of 29 or 20.68%) compared to those who did not (4 out of 85 or 3.4%) also resulted in statistically significant differences (X-squared = 5.05, df = 1, *p =* 0.02).

The types of greenspace within the 100 m buffers are presented below in Table 1. We further explored the ‘type’ of greenspaces around this 100 m radius and used Google Street View (GSV) as a manual confirmation tool. Following GSV public park or garden analysis, it was also discovered that in four of the 100 m buffers for GPs that did prescribe GRx, there were additional large greenspaces (public parks, *n* = 2; sports field, *n* = 1, and scrub/grassland, *n* = 1) not registered in the OS Open Greenspace dataset, and only one additional greenspace (sports field, *n* = 1) within 100 m of GPs that did not prescribe GRx (highlighted with asterisks). These additional greenspaces were included in the aforementioned analysis. 

**Figure 10:**
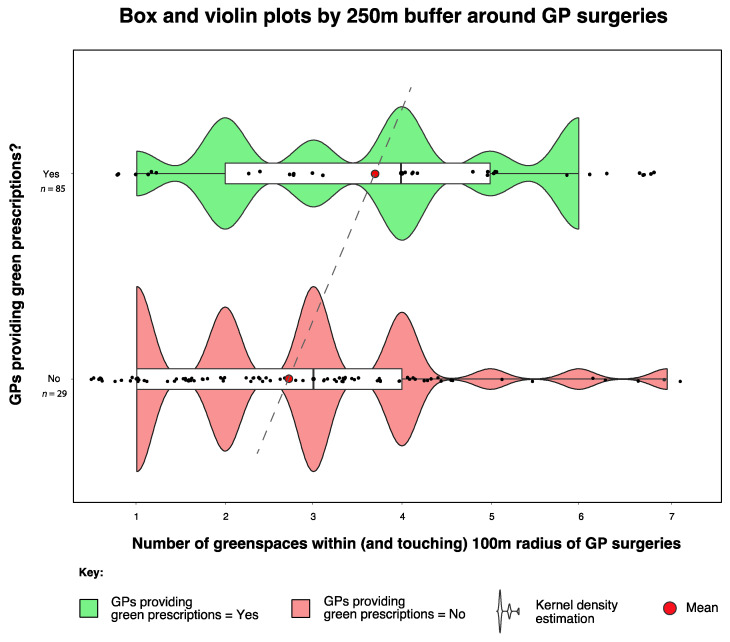
Box and violin plot showing differences in greenspace abundance within the 250 m buffer zone around GP surgeries that did (green) and did not (red) prescribe nature-based interventions.

A 2-sample test for equality of proportions confirmed that in terms of greenspace *presence* within a 250 m radius of GPs who prescribed nature-based interventions (23 out of 29 or 79.3%) compared to those who did not (69 out of 85 or 81.1%), there was no significant difference (X-squared = 1.78 × 10^−30^, df = 1, *p =* 1). However, we conducted the Wilcoxon rank sum test with continuity correction on the 250 m buffer and found that mean greenspace *abundance* within 250 m of group 1 (GPs prescribing nature-based interventions; $\bar{x}$ = 3.69) was significantly different (greater) from the same radius for group 2 (GPs not prescribing nature-based interventions; $\bar{x}$ = 2.74) (W = 524, *p =* 0.013) (Figure 10). Table 2 shows the abundance of greenspaces for all buffer radii between 100 m and 5000 m around GP surgeries.

Initial indications suggested that greenspace abundance was higher for the remaining radii. However, these failed to reach statistical significance. For example, greenspace abundance within 5 km of the GP surgeries that prescribed nature-based interventions ( $\bar{x}$ = 280) was higher compared to areas (within 5 km) where GP surgeries did not prescribe nature-based interventions ( $\bar{x}$ = 234). However, following a Wilcoxon rank sum test with continuity correction, these failed to reach statistical significance (W = 1044, *p =* 0.22).

**Figure 11:**
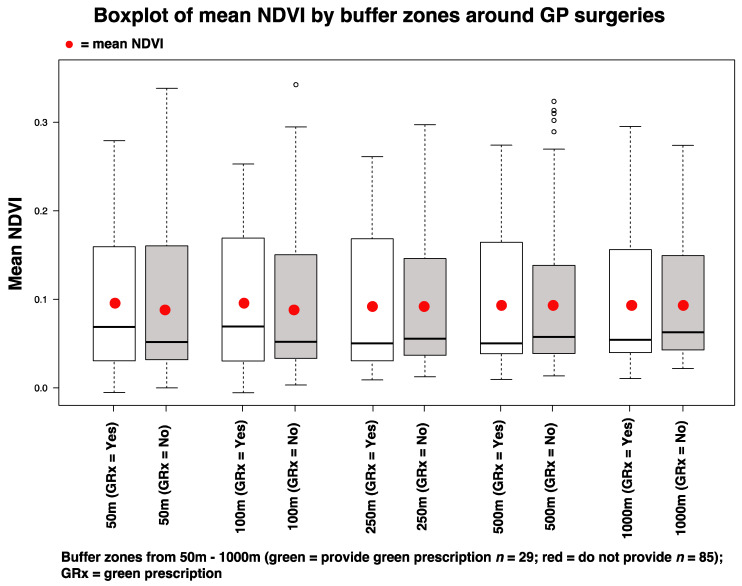
Boxplots showing mean NDVI scores for each buffer zone (50 m–1000 m) around GP surgeries that either did prescribe nature-based interventions (GRx = Yes) or did not (GRx = No).

For the NDVI analysis, the mean NDVI values (within 50 and 100 m buffer zones) where GPs prescribed nature-based interventions were higher ( $\bar{x}$ = 0.095 and $\bar{x}$ = 0.098, respectively) compared to the same radii where GPs did not prescribe nature-based interventions ( $\bar{x}$ = 0.085 and $\bar{x}$ = 0.086) (Figure 11). However, we generated a binomial logistic regression model for these parameters, and the differences were shown to be nonsignificant (GLM, *p* = 0.539 for 50 m; *p =* 0.497 for 100 m). 

#### 3.2.2. Deprivation Analyses

Mean IMD scores for areas (LSOAs) where GPs did prescribe GRx ($\bar{x}$ = 3.58) were higher than mean IMD scores for areas where GPs did not prescribe GRx ( $\bar{x}$ = 3.18). However, based on the results of a Wilcoxon rank sum test with continuity correction in R, these were not statistically significant (W = 1339, *p =* 0.1703). 

**Figure 12:**
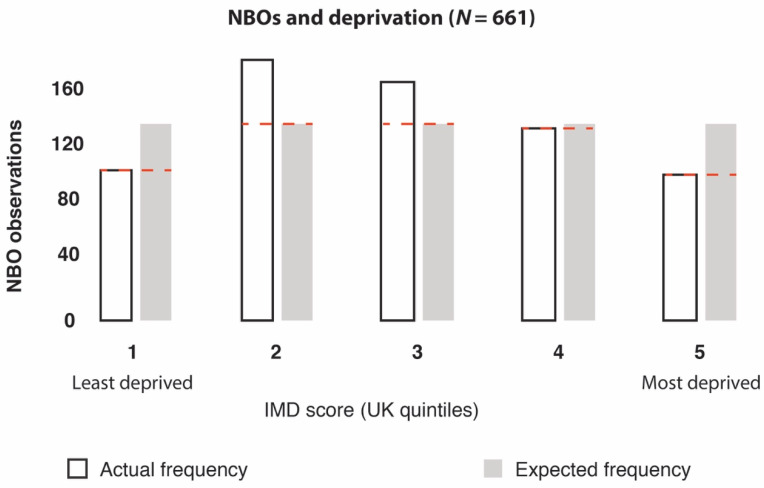
Output of *X*^2^ results: The frequencies of nature-based organisations (NBOs) were significantly different between areas with different levels of deprivation (based on UK IMD quintile scores), where 1 = least deprived and 5 = most deprived. Note, *n* = 53 NBO records contained zero IMD data.

**Figure 13:**
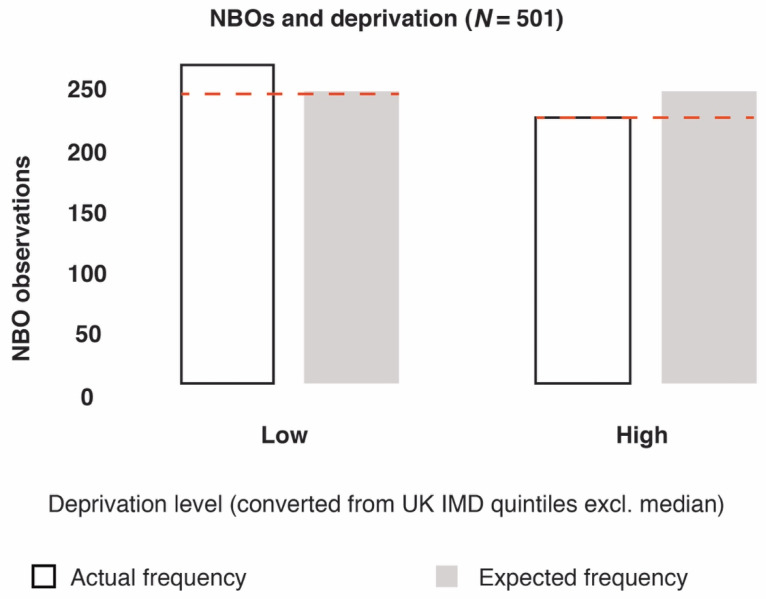
Output of *X*^2^ results: the frequencies of NBOs were significantly different between areas with low and high levels of deprivation (based on converting UK IMD quintile scores into low- and high-deprivation categories).

When analysing NBOs from the web-scrape (a combination of confirmed and unconfirmed GRx providers; *n* = 714), we found significant differences in the frequency of NBOs between areas with different levels of deprivation (*X*^2^ = 35.36, df = 4, *p =* 3.71966 × 10^−7^) (Figure 12). For sensitivity analysis, we also converted IMD quintile scores 1 and 2 into a “low” deprivation category, and quintile scores 4 and 5 into a “high” deprivation category, which confirmed statistically significant differences (*X*^2^ = 4.4, df = 1, *p =* 0.035) (Figure 13). This test calculated what the probability was that the difference in values (frequency of observations) was by chance under the assumption of independence.

#### 3.2.3. Geographical Presence of NBOs (Confirmed and Unconfirmed GRx Providers)

There were more likely to be NBOs who did provide GRx activities present within 5 km of GP surgeries that did prescribe nature-based interventions (14 out of 29 or 48.3%) compared to GP surgeries that did not prescribe nature-based interventions (22 out of 85 or 25.8%). This was confirmed by a 2-sample test for equality of proportions (X-squared = 4.0355, df = 1, *p=* 0.04455).

When including all NBO records acquired by the web-scrape (a combination of confirmed and unconfirmed providers; *n* = 714), the mean number of NBOs ( $\bar{x}$ = 2.7) within 5 km of GP surgeries prescribing nature-based interventions (*n* = 29) was greater than the mean number of NBOs within 5 km of GP surgeries not prescribing nature-based interventions ( $\bar{x}$ = 1.7; *n* = 85). However, this difference was not statistically significant (W = 1481, *p* = 0.09187). 

## 4. Discussion

In this study, we aimed to contribute to the growing but still limited knowledge base underlying green prescribing (i.e., prescribing nature-based health interventions) as a practical service. To this end, we mapped green prescribing services in the UK, explored spatial and socioecological relationships, and acquired the views from both GPs (as potential *prescribers*) and NBOs (as potential *providers*). 

A diverse suite of studies now supports the concept that spending time in nature can improve one’s health and well-being [58,59,60], and calls have been made to integrate nature-based and social prescribing into public health strategies [61,62,63]. There is also growing advocacy to support holistic integrative strategies such as green prescribing to enhance planetary health (through co-benefits to humans and the environment) [38,64,65]. However, there is limited understanding of the current status of (awareness and distribution), and socioecological relationships and constraints associated with green prescribing as a practical model of health care. An improved understanding of this could aid the optimization of management strategies and spur further research to overcome the constraints.

Our study confirms that green prescribing is active in numerous areas of the UK. We mapped some of the potential prescribers (GPs) and providers (NBOs) and acquired a diverse list of nature-based activities across the UK via a comprehensive web-scrape. With additional collaborative input, this latter process could form the basis of an expandable/editable database to allow primary health care professionals to search for local nature-based organisations and services that could support their patients. 

Our results suggest that GPs and NBOs perceived and expressed some common but also distinct constraints to green prescribing. Some of the common constraints included a shortfall of funding and time, and a lack of awareness of the green prescribing concept. The constraint most frequently expressed by GPs was not funding but the perceived lack of available services (i.e., organisations to support patients in engaging with interventions). Interestingly, a key constraint expressed by NBOs was the inability to engage with GPs and other primary care professionals. This disharmonic perception exemplifies the importance of establishing transdisciplinary collaborative pathways that are time efficient, and a common vocabulary in the area of green prescribing. Alongside the research that is needed to gain a greater understanding of the interventions themselves (as evidence may be lagging behind practice) [66,67], additional action is needed to improve the infrastructure management required to connect the different stakeholders (e.g., primary and social care, NBOs and patients) and to establish effective referral and monitoring processes—with personalised approaches in mind. In the UK, the recent formation of primary care networks (PCNs) (networks of practices that serve 30,000–50,000 patients)—and the provision of funding to employ ‘social prescribers’—could provide an important opportunity for early integration of green prescribing and could stimulate support for the additional research that is needed. 

It is widely accepted that greenspaces have an important role to play—ecologically and socially—in supporting personal, community and planetary health [68,69,70,71]. Furthermore, greenspaces are a fundamental resource (e.g., the archetypal setting) for GRx activities [72,73,74,75]. The significant association between greenspace presence and abundance within a 100 and 250 m radius of GP surgeries and the likelihood of providing green prescriptions was an interesting finding. This prompts a suite of additional questions such as: does the presence of local greenspaces influence the decisions by the GPs to prescribe GRx, or the decision by patients to enquire about GRx? Is the presence of greenspaces an indication of potential GRx activities in the area, and as such, does the availability of services equate to increased GRx provision and vice versa, i.e., does the lack of available services/infrastructure equate to limited GRx provision? Another of our findings suggests that significantly more NBOs were present within 5 km of GP practices that did prescribe GRx. This implies that the presence of available services could indeed affect the provision of GRx. However, further research is needed to verify this. Promisingly, collaborative networks involving medical authorities and nature-based organisations are increasing in presence and activity (e.g., the Centre for Sustainable Health care; www.sustainablehealth care.org.uk). Providing more support for these kinds of networks at a local scale would likely bring considerable value.

Other future pertinent questions include does surrounding greenspace influence the decision of eco-centric GPs (who may be more likely to prescribe GRx) to move to a given practice? Does the presence of greenspace reflect the socioeconomic status of an area, and does this increase the likelihood of GRx provision? And what element/s of the greenspace are important (e.g., size, type, quality, greenness, biodiversity)? We have made an initial contribution towards understanding this latter point—i.e., our results suggest that greenness (based on mean NDVI calculations for different buffer radii around GP surgeries) may not be a significant factor. Further research into the quality of greenspaces may be beneficial and there are several dimensions that could be explored, such as: maintenance, biodiversity, aesthetics, accessibility and the presence of facilities [52,76,77,78].

Studies have suggested that less deprived areas have a much higher prevalence of voluntary organisations than more deprived areas [79,80]. Considering that the majority of NBOs fall into the voluntary sector category, our results echo these previous studies and support the calls for governments, local authorities and also the NBOs themselves, to help secure ecological justice and provision of resources in areas of greatest need. 

Nonetheless, it is positive to see the initial indication of no significant differences between provision of GRx in areas of low and high deprivation—however, the small sample size calls for a cautionary approach to interpretation. Equitable access to high-quality greenspaces is likely to be important for personal and planetary health, and should therefore be a primary goal of health-centric urban policies [81]. If green prescribing is to play a key role in future health care strategies—alongside research that is needed to personalise these strategies—additional research into infrastructure management is needed to strengthen transdisciplinary collaborations. Further research into how local greenspace accessibility and quality may influence GRx would be beneficial, as would research that further scrutinises the equitable status of GRx resources. It could also prove valuable to explore the professional development experiences of prescribers and NBOs to identify their backgrounds and motivations—this could allow for a stronger indication of why and how their GRx strategies become successful. 

### Limitations

Our study has some important limitations to consider. For example, the relatively small sample size for the questionnaire element means that our findings should be interpreted with caution—particularly in the realm of representativeness (for both the significant and nonsignificant results). Our questionnaires did not reach all of the GP practices in the UK due to ethical and hierarchical issues, and the lack of a comprehensive list of contacts. Secondly, the results of our study are correlational and, as such, more conclusive evidence is required to infer causation for any of the findings. Thirdly, our list of NBOs from the web-scrape process is highly unlikely to be an exhaustive list of these organisations in practice. The records only represent NBOs that are sufficiently advertised (with appropriate search engine optimization, e.g., the inclusion of relevant keywords) and have an active web presence. We were unable to isolate the intended stakeholder for ‘awareness’ category in the questionnaire (i.e., whether this refers to GP, patients or both). There are several categories in the questionnaire results for perceived constraints that may have a degree of overlap—for example, “funding” and “resources” may overlap, as may “engaging GPs” and “lack of referrals”. However, these were considered to not significantly affect the interpretation the results. “Ecotherapy” is also a vague category from the web-scrape that could include several the other activities.

## 5. Conclusions

We have shown that green prescribing is happening in numerous parts of the UK. We created GIS outputs to highlight (based on the questionnaire results) the distribution of GPs that did prescribe nature-based interventions and the GPs that did not. We also plotted where NBOs facilitated green prescribing activities and where they did not, and we provided a comprehensive distribution map of NBOs (i.e., those with an online presence) via the web-scrape process. Our results suggest that GPs and NBOs perceive and express some common but also distinct constraints to green prescribing. Greenspace presence (but not greenness) and abundance within close proximity (100 and 250 m) to GP surgeries and NBO presence within 5 km were associated with higher levels of green prescribing provision. Lower levels of deprivation were associated with a higher frequency of NBOs but not with higher levels of green prescribing provision.

We hope that mapping green prescribing resources, acquiring views from GPs and NBOs, and conducting spatial/socioecological analyses will spur further research in this area. Establishing transdisciplinary collaborative pathways and a common vocabulary in the area of green prescribing would no doubt bring immense value, as would more research on personalised interventions. Action is needed to improve infrastructure management, particularly strategies that optimize stakeholder connectivity, referral mechanisms and monitoring processes. Further research into how local greenspace accessibility and quality may influence green prescribing could also bring value. Green prescribing has the potential to make an important contribution to personal and planetary health, but more support and research are needed to initiate, optimize and sustain these strategies. 

## Figures and Tables

**Table 1:** A list of greenspace type within a 100 m buffer radius of GPs that do and do not prescribe GRx.

Type of Greenspace	Number in 100 m of GRx = “Yes” *(n* = 29)	Number in 100 m of GRx = “No” *(n* = 85)
Playing field	5	6
Other sports facility	5	3
Play space	3	6
Cemetery	1	1
Allotment or community garden	3	5
Religious grounds	7	8
Public park or garden	6	10
Bowling green	1	1
Tennis court	1	1
Golf course	0	1
Public park *	2	0
Sports field *	1	1
Grassland/scrub *	1	0

* Additional greenspaces not registered in the OS Open Greenspace dataset.

**Table 2:** Greenspace abundance for all buffer radii between 100 m and 5 km (50 m excluded due to data deficiency) around GP surgeries.

Radius	Total Greenspaces	Mean	Median	Standard Deviation
100 m GRx Yes	34	1.17	1	1.12
100 m GRx No	85	0.51	0	0.81
250 m GRx Yes	85	3.69	4	1.66
250 m GRx No	188	2.72	3	1.49
500 m GRx Yes	239	8.24	8	3.80
500 m GRx No	554	6.50	6	3.50
1000 m GRx Yes	602	20.70	21	11
1000 m GRx No	1669	19.60	19	9
5000 m GRx Yes	8120	280.00	297	210
5000 m GRx No	19,936	234.00	190	209
